# Effect of the Coil Excitation Method on the Performance of a Dual-Coil Inductive Displacement Transducer

**DOI:** 10.3390/s23073703

**Published:** 2023-04-03

**Authors:** Jikang Xu, Yanchao Li, Ruichuan Li, Junru Yang, Xiaodong Yu

**Affiliations:** 1 College of Mechanical and Electronic Engineering, Shandong University of Science and Technology, Qingdao 266590, China; 10431220006@stu.qlu.edu.cn (J.X.); liruichuan@qlu.edu.cn (R.L.);; 2 College of Transportation, Shandong University of Science and Technology, Qingdao 266590, China

**Keywords:** dual-coil inductive displacement transducer, conditioning circuit, coil excitation method, linearity, sensitivity

## Abstract

A dual-coil inductive displacement transducer is a non-contact type measuring element for measuring displacement and is widely used in large power equipment systems such as construction machinery and agricultural equipment. However, the effect of the coil excitation method on the performance of dual-coil inductive displacement sensors has not been studied. This paper investigates the impact of different coil excitation methods on the operating performance of displacement transducers. The working principle, electromagnetic characteristics, and electrical characteristics were analyzed by building a mathematical model. A transducer measurement device was used to determine the relationship between core displacement and coil inductance. Three coil excitation methods were proposed, and the effects of the three coil excitation methods on the amplitude variation, phase shift, linearity, and sensitivity of the output signal were studied by simulation based on the AD630 chip as the core of the conditioning circuit. Finally, the study’s feasibility was demonstrated by comparing the experiment to the simulation. The results show that, under the uniform magnetic field strength distribution in the coil, the coil voltage variation is proportional to the inductive core displacement. The amplitude variation is the largest for the dual-coil series three-wire (DCSTW) and is the same for the dual-coil series four-wire (DCSFW) and dual-coil parallel differential (DCPD). DCSFW has an enormous phase shift. DCSTW has the best linearity. The research in this paper provides a theoretical basis for selecting a suitable coil excitation, which is conducive to further improving the operating performance of dual-coil inductive displacement transducers.

## 1. Introduction

Displacement transducers are widely used in construction machinery, agricultural equipment, and other large power equipment systems, for which inductive displacement transducers are the ideal choice. Inductive displacement transducers are characterized by non-contact, high linearity, and low price. The moving part of the transducer does not require an electrical connection and is, therefore, more reliable [1]. Inductive transducers are divided into different types, such as variable reluctance, mutual inductance and eddy current. Different types of transducers have different application scenarios. Still, they all convert the measured physical quantity into the change of coil inductance. Then, the conditioning circuit converts the change of inductance into the change of voltage or current to realize the conversion of non-electricity to electricity.

Inductive displacement transducers have a simple structure and operate with a non-uniform internal magnetic field, so the relationship between the detected quantity and the output signal is not ideally linear. The structure, excitation, and conditioning circuits can be optimized to obtain better operating performance. Ren et al. proposed a high-performance signal processing circuit for a three-coil inductive displacement transducer [2]. A sinusoidal voltage signal is applied to both coils, and a conditioning circuit demodulates the output signal of the middle coil. An unbalance compensation circuit is added to increase the stability and accuracy of the conditioning circuit’s output signal. Sandra et al. studied an inductive displacement transducer consisting of a circuit board planar coil and an E-type soft iron core [3]. The movement of the plane coil causes a change in inductance, and the inductance value characterizes the displacement. Grima et al. proposed a coreless inductive displacement transducer [4,5]. The transducer consists of two supply coils, two induction coils, and a moving coil. The supply coil and induction coil are wrapped around the fixed tube in two layers. The effects of excitation signal frequency and different types of sense cores on the output signal were investigated. Dian Jiao et al. investigated the effect of different gaps and different materials of metal target plates on the transducer performance of eddy current transducers [6]. Mirzaei et al. studied the effect of different temperatures and different ferromagnetic materials on inductive position transducers [7]. The thermal stability of the sensor was analyzed, and a compensation scheme was proposed. Liu and Bu used a conditioning circuit based on the AD598 chip. The chip can output both the sinusoidal excitation signal and the demodulated output signal [8], and the effectiveness of the conditioning circuit has been verified through experiments. Reverter and Gasulla studied a timer-based AM demodulator [9]. The dual-coil inductive displacement transducer was applied with a sinusoidal voltage excitation signal, and the connection point of the two series coils was used as the output signal. The signal was demodulated by the AM demodulator.

As seen above, the effect of the coil excitation method on the performance of inductive displacement transducers was not studied. Since different coil excitation methods have different effects on the performance of inductive displacement sensors, choosing the appropriate coil excitation is critical to the performance of transducers.

Based on the dual-coil inductive displacement transducer, this paper analyzes its working principle and characteristics. It adopts a conditioning circuit with an AD630 chip as the core, which can realize the demodulation of the transducer’s output signal. Three coil excitation methods are proposed, and the principles of the three excitation methods are analyzed. The effect of different excitation methods on the transducer performance is studied by simulation, and the simulation results are analyzed. Finally, it is verified by experiments. The research results provide a foundation for selecting the appropriate coil excitation for inductive displacement transducers.

## 2. Structure and Operating Principle of a Dual-Coil Inductive Displacement Transducer

### 2.1. Structure of Dual-Coil Inductive Displacement Transducer

**Figure 1:**
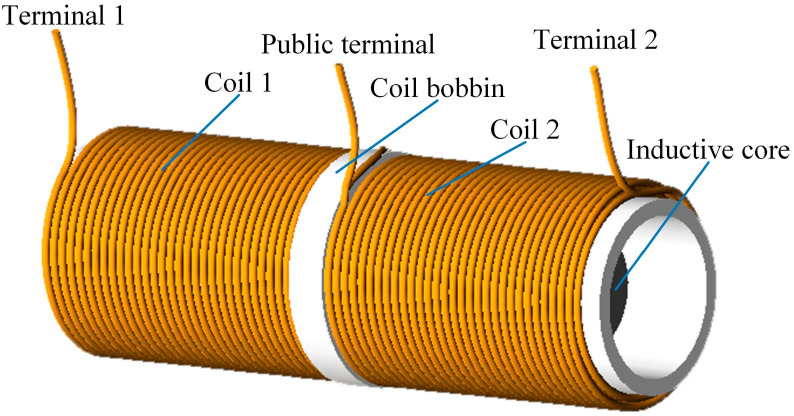
Structure of a dual-coil inductive displacement transducer.

Dual-coil inductive displacement transducers consist mainly of two coils connected in series, a coil bobbin and an inductive core. The two series coils are perfectly symmetrical, the coil skeleton is magnetically isolated, and the inductive core has good permeability. The dual-coil inductive displacement transducer studied in this paper has three terminals. The two coil connection nodes are the public terminal, and the other ends of the wire section are terminal 1 and terminal 2, respectively. A sketch of the structure is shown in Figure 1.

### 2.2. Principle of Operation

**Figure 2:**
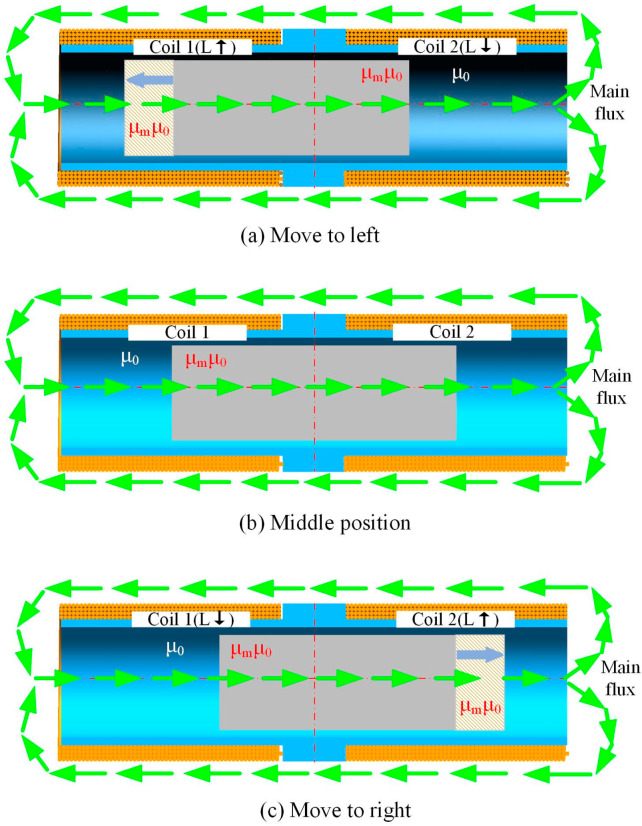
Operating principle of a dual-coil inductive displacement transducer. In the figure, 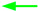 Represents the direction of magnetic flux, 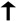 Represents an increase in inductance, 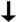 Represents a reduction in inductance.

Dual-coil inductive displacement transducers operate with two identical series-connected coils and an internal inductive core. When the displacement transducer is in operation, the magnetic permeability of the hollow coil is *µ*_0_, and the relative permeability of the inductive core is *µ_m_*, so the magnetic permeability of the inductive core is *µ*_0_*µ_m_*. The principle of operation of a dual-coil inductive displacement transducer is shown in Figure 2. The green arrow in the diagram represents the main magnetic circuit; the central moving part is the inductive core; the yellow part is the coil; and the blue part is the coil bobbin. The axial length of both coils is *a*, the number of turns per unit length of the coil is *n*, and the cross-sectional area of the coil is S.

Coil inductance is calculated as

(1) $$L=\frac{\Phi }{I}=\frac{a\int BdS}{I}=\frac{a\int \mu nIdS}{I}=\frac{a\mu nIS}{I}=a\mu nS$$

where *L* is the coil inductance, Φ is the coil flux, *I* is the current in the coil, *B* is the strength of the magnetic field in the coil, µ is the magnetic permeability, *a* is the axial length of coils, and *n* is the number of turns per unit length of the coil.

According to Equation (1), it can be seen that the inductance of a dual-coil inductive displacement transducer is related to the magnetic permeability within the coil. When the inductive core is in the middle position, as shown in Figure 2b, the left and right sides of the transducer are symmetrical in the structure; the inductive cores are inserted into the coils at the same length. Therefore the permeabilities of coils 1 and 2 are the same, and both coils have the same inductance. The inductive core is moved from the middle to the left, as shown in Figure 2a. The length of the inductive core inserted into coil 1 increases, and the permeability increases. The length of the inductive core inserted into coil 2 decreases, and the permeability decreases. As a result, the inductance of coil 1 increases and the inductance of coil 2 decreases. At this point, the inductance of coil 1 is greater than the inductance of coil 2. The inductive core is moved from the middle to the right, as shown in Figure 2c. The length of the inductive core inserted into coil 1 decreases, and the permeability decreases. The length of the inductive core inserted into coil 2 increases, as does the permeability. As a result, the inductance of coil 1 decreases and the inductance of coil 2 increases. At this point, the inductance of coil 1 is less than that of coil 2. The essence of the working principle of a dual-coil inductive displacement transducer is the change in the coil’s inductance caused by a difference in the position of the inductive core. The conditioning circuit converts the change in inductance into a change in voltage or current, enabling the displacement to be measured.

### 2.3. Electromagnetic Characteristic Analysis

**Figure 3:**
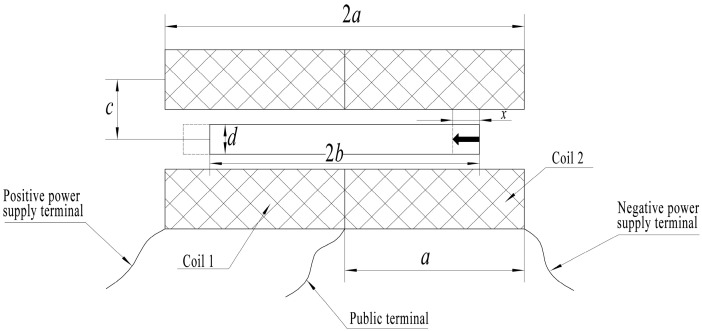
Schematic diagram of the structure of a dual-coil inductive displacement transducer.

The inductive core of the displacement transducer is rigidly connected to the object to be measured, with the inductive core moving to the left as an example, as shown in Figure 3 [10]. The position of the object to be measured is obtained by measuring the position of the inductive core. After the coil has applied excitation, assume that the inductive core moves *x* to the left [11]. The two coils are equal in axial length and have *a* radius of *c*. The inductive core is located in the middle of the two coils, and the length and diameter of the inductive core are *2b* and *d*, respectively.

Two coils are connected in series. Each coil’s magnetic chain consists of two parts: the magnetic chain generated by the hollow coil and the magnetic chain generated by the coil after the insertion of the inductive core.

According to the Biot–Savart law, the axial magnetic induction intensity of a hollow coil is [12,13]:

(2) $$B=\frac{\mu_{0}nI}{2}\left(\frac{x+a}{\sqrt{{\left(x+a\right)}^{2}+c^{2}}}-\frac{x-a}{\sqrt{{\left(x-a\right)}^{2}+c^{2}}}\right)$$

Equation (2) represents the axial component of the magnetic induction, which is related to the coil length *a* and the coil radius *c*. When *a* ≫ *c*, Equation (2) can be simplified to

(3) $$B\approx \mu_{0}nI$$

The hollow coil inductance *L*_0_ is

(4) $$L_{0}=a\mu_{0}nS=a\mu_{0}n•\pi •c^{2}=\pi a\mu_{0}nc^{2}$$

When the inductive core is inserted into the hollow coil, the permeability of a part of the coil increases from *µ*_0_ to *µ*_0_*µ_m_*. As the permeability increases, the inductance increases. The inductance *L_m_* within the inductive core is

(5) $$L_{m}=\pi a\mu_{0}\mu_{m}nc^{2}$$

When the inductive core is in the middle position, coil 1 inductance *L_mid__*_1_ and coil 2 inductance *L_mid__*_2_ are equal, both being *L_mid_*. The expression is

(6) $$L_{mid}=L_{mid\_1}=L_{mid\_2}=\pi a\mu_{0}nc^{2}(a-b)+\pi a\mu_{0}\mu_{m}nc^{2}b$$

When the inductive core is moved *x* to the left, the inductance values *L_left_*____1_ of coil 1 and the inductance value *L_left_*____2_ of coil 2 are

(7) $$L_{left\_1}=\pi a\mu_{0}nc^{2}(a-b-x)+\pi a\mu_{0}\mu_{m}nc^{2}(b+x)$$

(8) $$L_{left\_2}=\pi a\mu_{0}nc^{2}(b+x)+\pi a\mu_{0}\mu_{m}nc^{2}(a-b-x)$$

The variation of the inductance of coil 1 ∆*L_left__*_1_ [14] is

(9) $$\Delta L_{left\_1}=L_{left\_1}-L_{mid}=(\mu_{m}-1)\pi a\mu_{0}nc^{2}x$$

The variation of the inductance of coil 2 ∆*L_left__*_2_ is

(10) $$\Delta L_{left\_2}=L_{left\_2}-L_{mid}=-(\mu_{m}-1)\pi a\mu_{0}nc^{2}(2b-a+x)$$

Equations (9) and (10) show that the inductance of coil 1 increases, and the inductance of coil 2 decreases after the inductive core is moved to the left. Since the structure of the dual-coil inductive displacement transducer is symmetrical, the increase in inductance of coil 1 and the decrease in inductance of coil 2 are equal, therefore

(11) $$\Delta L_{left\_1}=-\Delta L_{left\_2}$$

Equation (11) holds if the length of the solenoid coil is infinite. For a finite-length solenoid coil, the magnetic field strength at the end of the solenoid coil is smaller than the center position. When the sensing core moves to the left, the increased inductance of coil 1 is smaller than the reduced inductance of coil 2.

From this, it follows that

(12) $$b=\frac{a}{2}$$

Therefore, the length of the inductive core is equal to the length of the individual coils.

It can be seen from Equation (9) that, under conditions of uniform distribution of magnetic field strength in the coil, the change in inductance value ∆*L* is proportional to the amount of change in the position of the inductive core *x*. In practice, due to the limited length of the coil, the magnetic field strength in the coil is not uniformly distributed, and the relationship between input and output is non-linear.

### 2.4. Electrical Characteristic Analysis

**Figure 4:**
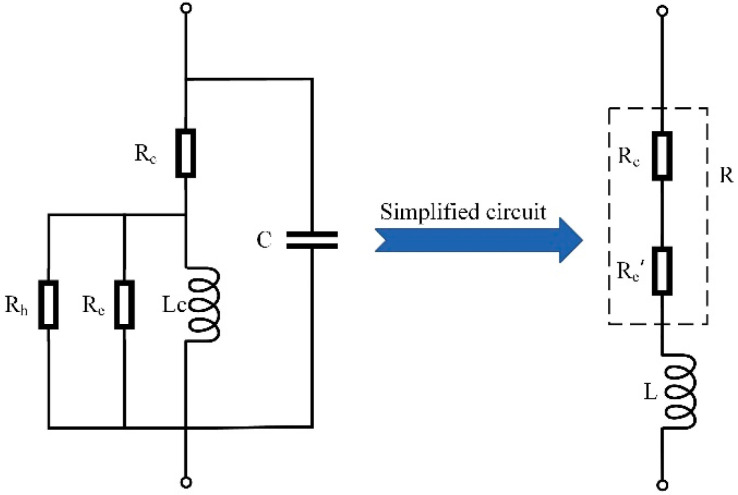
Displacement transducer single coil equivalent circuit.

The coil of a dual-coil inductive displacement transducer is not an ideal purely inductive coil. In addition to inductance *L_c_*, it includes copper loss resistance *R_c_*, core eddy current loss resistance *R_e_*, hysteresis loss resistance *R_h_*, and parallel parasitic capacitance *C* [15]. For analysis convenience, ignoring the parallel parasitic capacitance *C*, the inductive coil and its parallel resistance are equivalent to the iron-loss resistance *R_e_*’ and inductance *L*; the copper-loss resistance and iron-loss resistance are regarded as the resistance *R* of the inductive coil. The actual and simplified equivalent circuits are shown in Figure 4.

**Figure 5:**
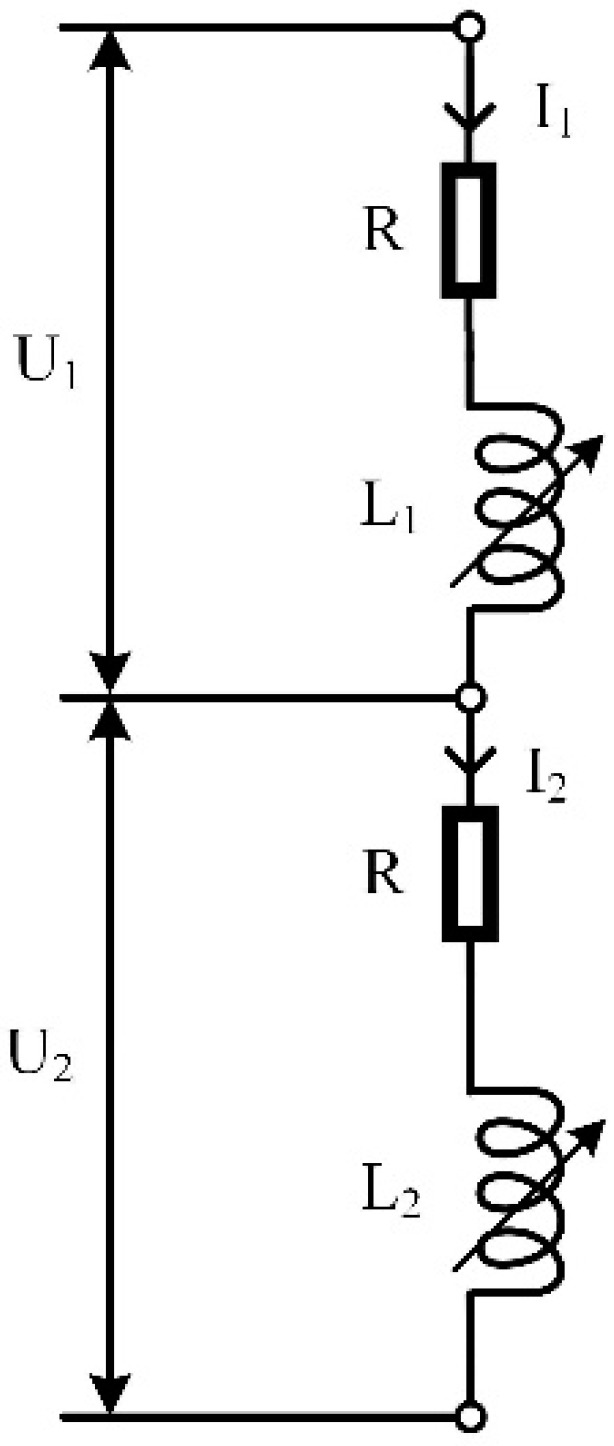
Equivalent circuit of a dual-coil inductive displacement transducer.

When the dual-coil inductive displacement transducer works, the effect of inductive core displacement on coil resistance is minimal and negligible. The dual-coil inductive displacement transducer converts the position change into an inductance change. The inductance of coil 1 is *L*_1_, the current is *I*_1_, and the voltage is *U*_1_. The inductance of coil 2 is *L*_2_, the current is *I*_2_, and the voltage is *U*_2_. The resistance of both coils is *R*. The equivalent circuit of the dual-coil inductive displacement transducer is shown in Figure 5 [16].

The impedance *Z*_1_ of coil 1 and the impedance *Z*_2_ of coil 2 in Figure 5 can be calculated using the following [17,18]

(13) $$Z_{1}=R+j\omega L_{1}$$

(14) $$Z_{2}=R+j\omega L_{2}$$

Assume that the currents in coils 1 and 2 are equal and are I. Then

(15) $$U_{1}=Z_{1}I=(R+j\omega L_{1})I$$

(16) $$U_{2}=Z_{2}I=(R+j\omega L_{2})I$$

When the inductive core is in the middle position, the voltage of coil 1 is *U_mid__*_1_, the voltage of coil 2 is *U_mid__*_2_, and both voltages are *U_mid_*, which can be expressed as

(17) $$U_{mid}=U_{mid\_1}=U_{mid\_2}=(R+j\omega L_{mid})I$$

After the inductive core has moved *x* to the left, the voltage of coil 1 is *U_left__*_1_, and the voltage of coil 2 is *U_left__*_2_, which can be expressed as

(18) $$U_{left\_1}=(R+j\omega L_{left\_1})I$$

(19) $$U_{left\_2}=(R+j\omega L_{left\_2})I$$

After the inductive core moves to the left, the voltage variation ∆*U_left__*_1_ of coil 1 and the voltage variation ∆*U_left__*_2_ of coil 2 [19,20] are

(20) $$\Delta U_{left\_1}=U_{left\_1}-U_{mid}=j\omega I\Delta L_{left\_1}$$

(21) $$\Delta U_{left\_2}=L_{left\_2}-L_{mid}=j\omega I\Delta L_{left\_2}$$

According to Equation (11), the variation ∆*U* of the coil voltage is

(22) $$\Delta U=\Delta U_{left\_1}=-\Delta U_{left\_2}$$

Equations (20) and (21) show that the voltage variation ΔU of the coil is proportional to the inductance variation ΔL. With a uniformly distributed magnetic field, the inductance variation is proportional to the inductive core position variation *x*, so the coil voltage variation is also proportional to the inductive core position variation.

### 2.5. The Principle of the Conditioning Circuit

**Figure 6:**
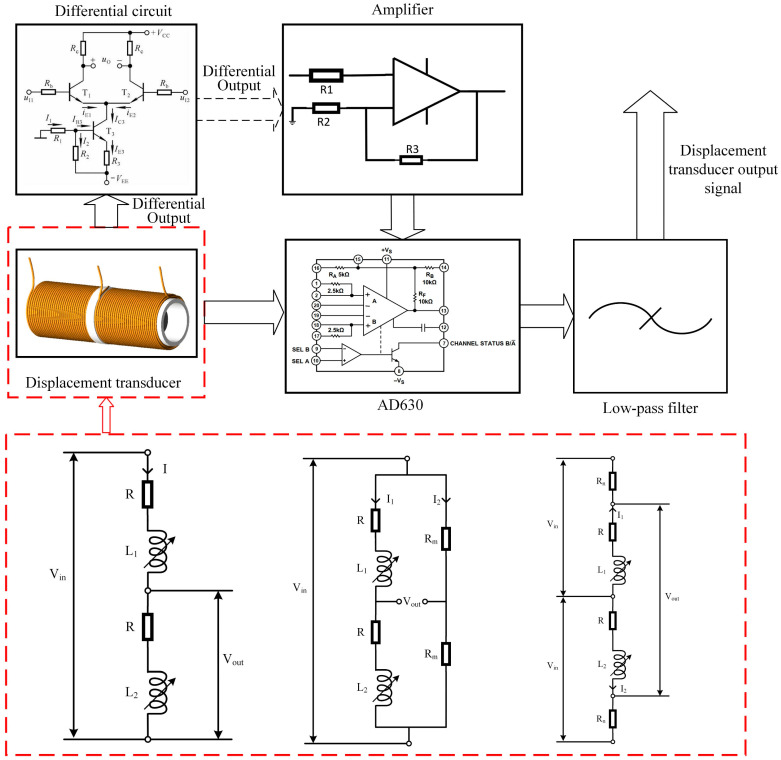
Block diagram of the conditioning circuit of a dual-coil inductive displacement transducer.

**Figure 7:**
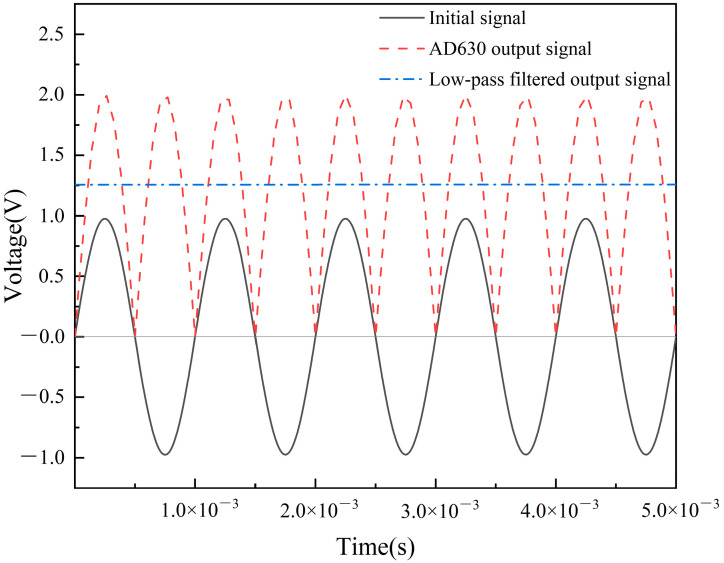
Operating principle of the AD630 signal conditioning circuit under a sinusoidal excitation signal.

The excitation applied to a dual-coil inductive displacement transducer is an AC signal, so the transducer’s original output signal needs to be demodulated using a suitable conditioning circuit. When a voltage excitation is applied to a displacement transducer, the output voltage amplitude changes as the position of the inductive core changes. To obtain the relationship between the inductive core displacement and the output signal, a conditioning circuit based on the AD630 balanced conditioner/demodulator was used [21]. First, the displacement transducer output signal was amplified using an operational amplifier, then it was processed by the AD630 circuit, and finally, a low-pass filter was used to obtain the DC signal [22]. If the displacement transducer is connected differentially [23], adding a differential circuit in front of the amplifier circuit is necessary to process the output signal. The block diagram of the conditioning circuit structure is shown in Figure 6. When the input initial signal amplitude is 1 V, the frequency is 1 kHz, and a square wave modulated signal of the same frequency and phase is used, the signal conditioning circuit demodulation principle is shown in Figure 7.

Figure 6 shows that the initial sinusoidal voltage signal is converted to a half-wave positive voltage signal with a voltage amplitude of 2 V after passing through the AD630 chip. After the low-pass filter, the voltage amplitude is 1.257 V. In the AD630 conditioning circuit, the frequency and phase of the modulating signal significantly affect the output signal. In Figure 7, the modulating signal has the same frequency as the input signal, and the phase difference is 0° when the output signal is a positive half-wave voltage. When the phase difference between the modulating signal and the input signal is 180°, the output signal is a negative half-wave voltage.

## 3. Excitation Method for Dual-Coil Inductive Displacement Transducers

According to the electromagnetic and electrical characteristics of the dual-coil inductive displacement transducer, the detection of the inductive core position can be achieved as long as the output signal is guaranteed to vary with the inductive core position. As a result, three coil incentives are proposed.

### 3.1. Dual-Coil Series Excitation Method

There are two types of dual-coil series excitation: a three-wire connection [24] and a four-wire connection [25].

**Figure 8:**
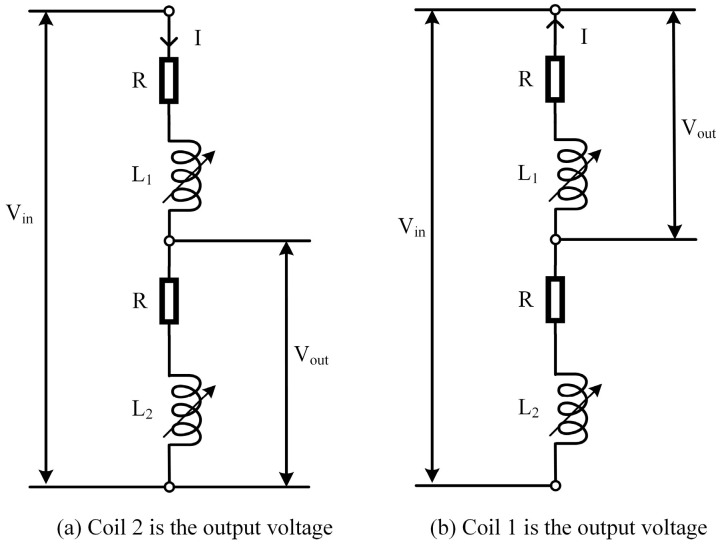
DCSTW excitation method.

The dual-coil series three-wire (DCSTW) excitation method applies an excitation signal to both ends of the transducer, and the public terminal and the negative terminal of the excitation signal are used as outputs [26], as shown in Figure 8. Vin is the excitation signal, and Vout is the output signal. When the inductive core is displaced, the coil’s magnetoresistance changes and the inductive core’s displacement is characterized by detecting the voltage change in one of the coils. The inductive core is moved from one end to the other within the stroke, and the coil’s output voltage varies within a specific range. Figure 8a detects the voltage of coil 2, and Figure 8b detects the voltage of coil 1. The two excitation methods have opposite voltage polarity and opposite current directions.

**Figure 9:**
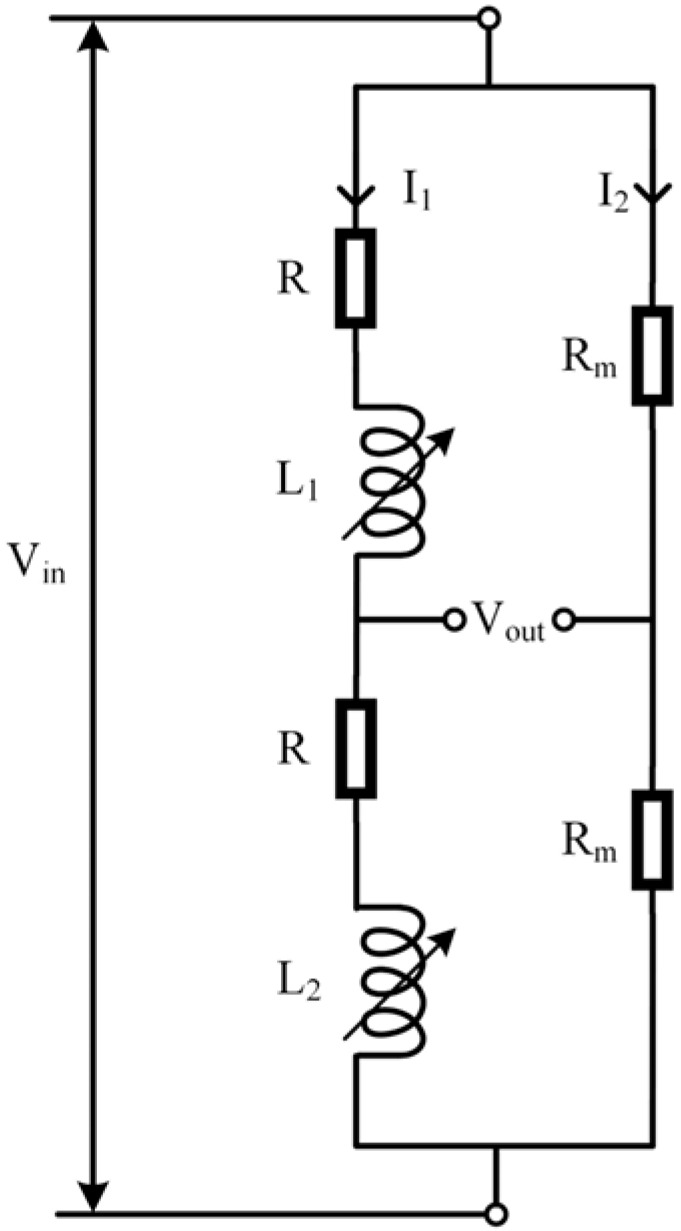
DCSFW excitation method.

The dual-coil series four-wire (DCSFW) excitation method is a differential bridge connection [27,28], where two matching resistors *R_m_* are connected in series and then in parallel with two series coils. This is a common excitation method today. The two terminals of the parallel circuit are the excitation signal inputs, and the public terminal of the transducer and the public terminal of the two series resistors *R_m_* are the signal outputs, as shown in Figure 9. *I*_1_ is the current flowing through the coil, and *I*_2_ is the current flowing through the matching resistor. The bridge is in equilibrium when the inductive core is in the middle position and the output voltage *V_out_* is 0 V. As the inductive core moved to coil 1, the voltage of coil 1 increased, and the voltage of coil 2 decreased. The voltage on the right side of *V_out_* was higher than on the left side, and the current direction was leftward. As the inductive core moved to coil 2, the voltage of coil 1 decreased, and the voltage of coil 2 increased. The voltage on the left side of *V_out_* was higher than the voltage on the right side, and the current direction was rightward. This circuit is used to characterize the displacement of the inductive core by comparing the voltage difference at the junction of the parallel branches. If a positive voltage is an output when the inductive core moves to coil 1, a negative voltage is an output when the inductive core moves to coil 2. This circuit not only measures the displacement magnitude but also determines the direction of displacement. To ensure the bridge is balanced, *Z*_1_ = *Z*_2_ = *Z*, where *Z*_m_ is the impedance of *R*_m_. The resistance value of the matching resistor is determined by [29]

(23) $$R_{m}=Z_{m}=Z_{1}=Z_{2}=R+j\omega X_{L}=\sqrt{R^{2}+{\left(2\pi fL_{mid}\right)}^{2}}$$

where *X_L_* is the inductive resistance of the coil and *f* is the frequency of the excitation voltage signal.

### 3.2. Dual-Coil Parallel Excitation Method

**Figure 10:**
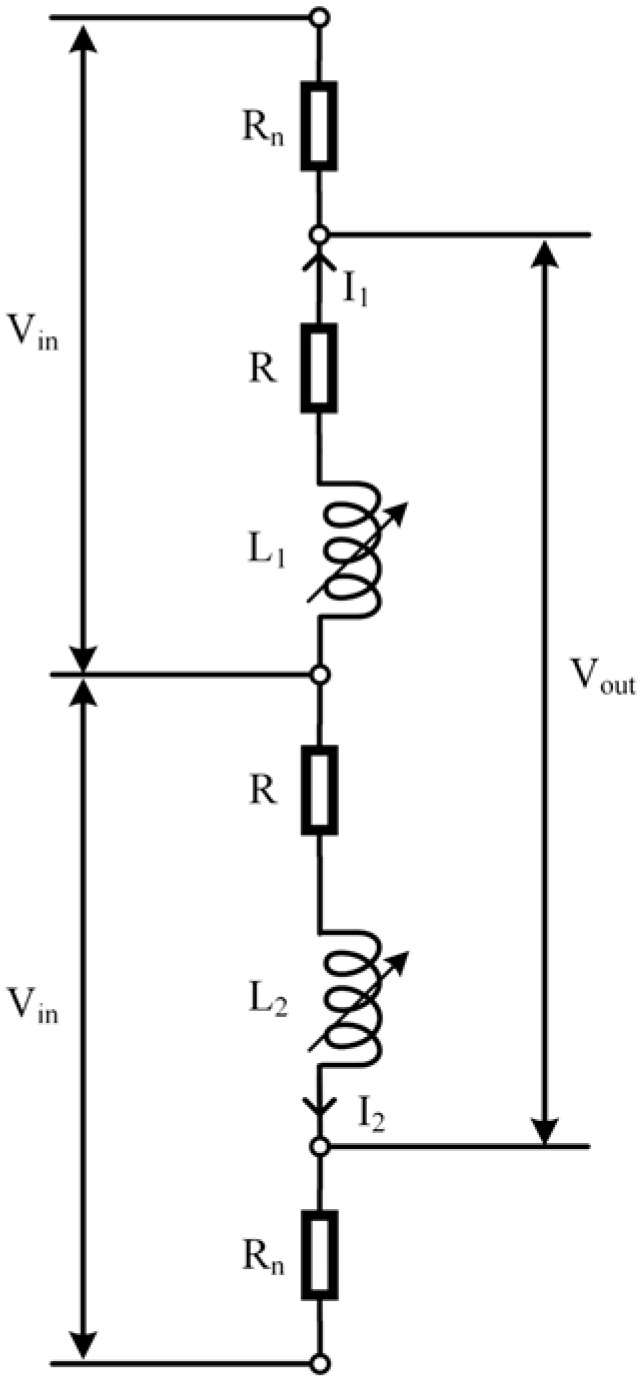
DCPD excitation method.

The dual-coil parallel differential (DCPD) excitation method was given to two coils in series with a resistor R_n_. The public terminal of the displacement transducer was the excitation signal input, and the junctions of the two resistors R_n_ and the coils were used as the signal output, where R_n_ = R, as shown in Figure 10. When the inductive core was in the middle position, the impedance of both branches was equal: *I*_1_ = *I*_2_. The voltage of the upper resistor Rn was equal to the voltage of the lower resistor Rn. The output voltage in this position was 0 V. As the inductive core moved to coil 1, the impedance increased, and the current *I*_1_ decreased in coil 1, while the impedance decreased and the current *I*_2_ increased in coil 2. Therefore, the voltage of the upper resistor was less than that of the lower resistor. As the inductive core moved to coil 2, the impedance decreased, and the current *I*_1_ increased in coil 1, while the impedance increased and the current *I*_2_ decreased in coil 2. Therefore, the voltage of the upper resistor was greater than that of the lower resistor. This circuit is used to characterize the displacement of the inductive core by detecting the difference in voltage between the two resistors. If a positive voltage is an output when the inductive core moves towards coil 1, a negative voltage is an output when the inductive core moves towards coil 2. Therefore, the circuit can also measure the magnitude and direction of the displacement.

## 4. Effect of the Coil Excitation Method on the Performance of Displacement Transducers

### 4.1. Basic Data Testing of Dual-Coil Inductive Displacement Transducers

**Figure 11:**
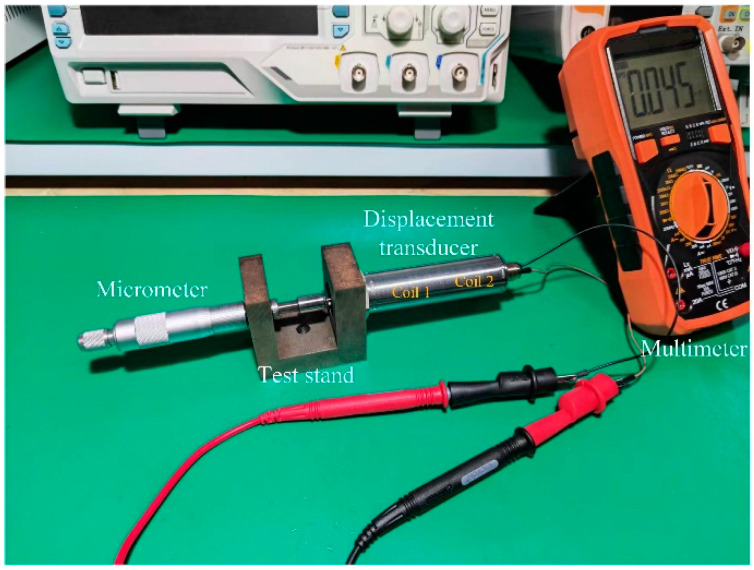
Testing of the resistance and inductance of DIPT16.

In this study, a dual-coil inductive displacement transducer, DIPT16, was used. To investigate the effect of different excitation methods on the output performance of this transducer, Multisim software was used to simulate the three excitation methods. Since the coil of the displacement transducer is equated to inductance and resistance in series, it is necessary to test the resistance and inductance of the coil in DIPT16. A displacement transducer test device was made, and a multimeter was used to test the resistance and inductance, as shown in Figure 11.

**Figure 12:**
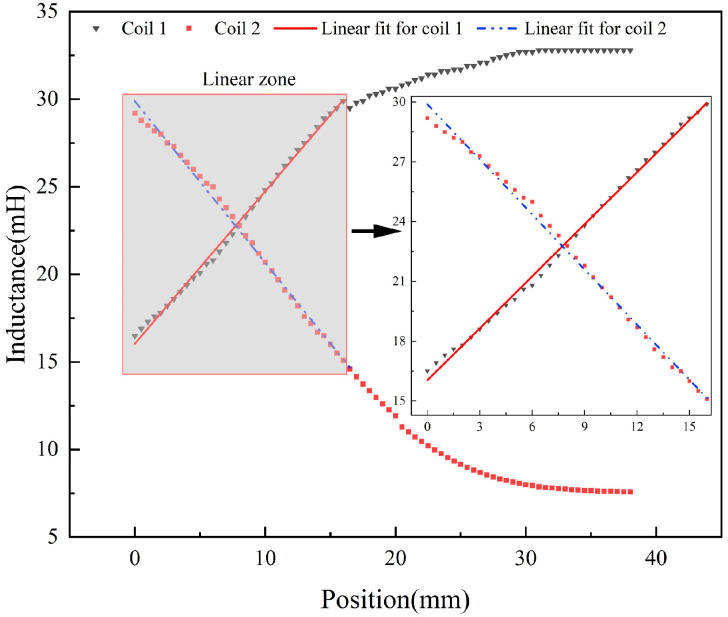
The relationship between coil inductance and core position in DIPT16.

The change in displacement of the inductive core was measured by a micrometer and a multimeter was connected to the two terminals of the coil for testing [30,31]. The initial position of the inductive core was at the rightmost end of coil 2, noted as 0 mm. During the measurement, the inductive core moved to coil 1, and the resistance and inductance were measured every 0.5 movements. The test found that the resistance of the coils remained constant as the inductive core moved, and the resistance of each coil was 94 Ω. Tests were carried out within a 38 mm movement of the inductive core. The change in inductance of the two coils is shown in Figure 12, and the linear fit results are shown in Table 1. As can be seen from the graph, the inductance variation from 0–16 mm is relatively uniform and linear. This range was chosen as the effective travel of the displacement transducer. A linear fit was made to the test data. Coil 1 had a Pearson’s r value of 0.99856, an Adj. R-Square value of 0.99703 and a mean square value of 0.05264. Coil 2 had a Pearson’s r value of 0.99792, an Adj. R-Square value of 0.99572 and a mean square value of 0.09048. It can be seen that the linearity of the inductance of coil 1 is better than coil 2 [32]. On the whole, the DIPT16 has better inductive characteristics.

### 4.2. Effects of the Excitation Method on Displacement Transducer Performance

The three coil excitation methods of the dual coil inductive displacement transducer all use the AD630 chip as the core signal conditioning circuit, and the modulating signal is a square wave signal that is the same frequency and phase as the excitation signal. The amplifier in the conditioning circuit has an amplification of 11. The series resistance *R_n_* of the two coils in DCPD is 100 Ω, and the two resistances *R_m_* of DCSFW in parallel are 171 Ω. The DCPD and DCSFW require additional differential circuitry in front of the operational amplifier. The differential circuit in DCPD allows the resistive voltage of coil 1 in series to be subtracted from the resistive voltage of coil 2 in series, and the differential circuit in DCSFW allows the voltage of the public terminal of the displacement transducer to be subtracted from the node voltage of the two series resistors.

**Figure 13:**
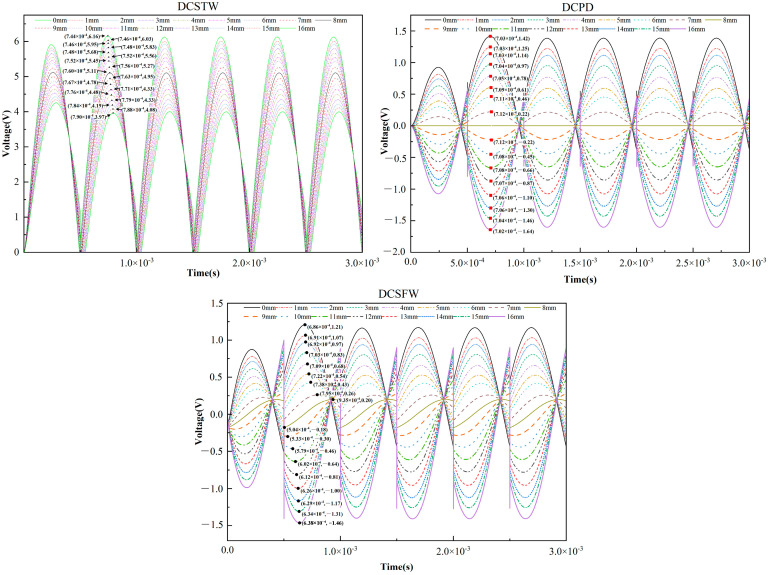
Comparison of the output signal of AD630 circuit under three excitation methods (before filtering).

When a sinusoidal signal is used as the excitation signal, the amplitude is 0.5 V, and the frequency is 1 kHz. The inductive core was recorded for every 1 mm movement [33]. Using the above three excitation methods, the displacement transducer’s original signal was processed by the AD630 circuit, and the output signal is shown in Figure 13.

**Figure 14:**
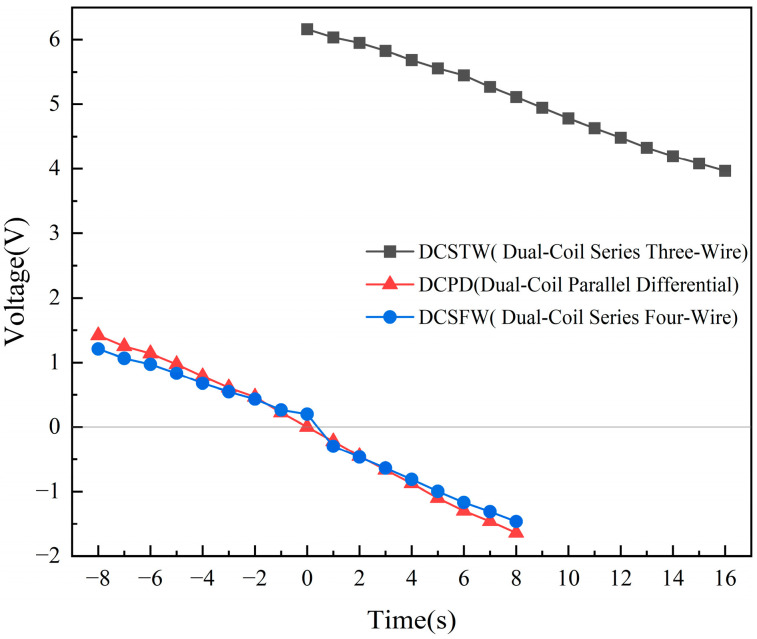
Variation in the amplitude of the output signal of the AD630 circuit.

**Figure 15:**
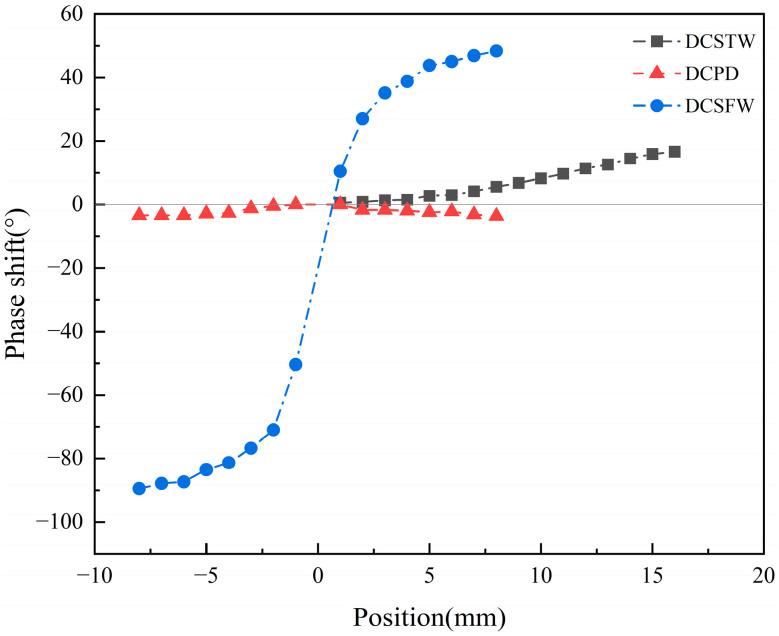
Phase shift of the output signal of the AD630 circuit.

As seen in Figure 13, there are differences in the amplitude change and phase shift of the output signal for the three excitation methods under sinusoidal signal excitation. Assume that the movement of the inductive core toward coil 1 is positive. For single-direction displacement measurement, the initial position is the rightmost end of coil 2. For bi-directional displacement measurement, the initial position is the middle position. During the change of inductive core displacement, the variation of the output signal amplitude of the AD630 circuit is shown in Figure 14, and the phase variation is shown in Figure 15.

As seen in Figure 14, the DCSTW can only provide a single-direction displacement test, while the DCPD and DCSFW can complete a bi-directional displacement test. The amplitude variation of DCSTW is 2.19 V. The variation in amplitude of the positive displacement of the DCPD is 1.42 V, the variation in amplitude of the negative displacement is 1.64 V, and the average variation is 1.53 V. The variation in amplitude of the positive displacement of the DCSFW is 1.21 V, the variation in amplitude of the negative displacement is 1.46 V, and the average variation is 1.34 V. The variation in amplitude of DCSTW is 1.43 times greater than DCPD and 1.63 times greater than DCSFW. The variation in amplitude of the DCPD is slightly greater than the DCSTW. As a result, single-direction displacement detection has a significant amplitude variation and high resolving power. DCPD and DCSFW have roughly the same amplitude variation, with DCPD having the higher resolving power.

As shown in Figure 15, the DCSTW has a positive phase shift, and the more significant the inductive core displacement, the greater the phase shift, where the maximum phase shift is 16.67°. In the inductive core bi-directional displacement, the phase shifts of the DCPD are all negative, of which the maximum phase shift is −3.60° for the positive displacement and −3.36° for the negative displacement. The DCSFW has a positive phase shift of positive displacement with a maximum phase shift of 48.38° and a negative phase shift of negative displacement with a maximum phase shift of −89.48°. This shows that the DCPD has the smallest phase shift, and the DCSFW has the most significant phase shift. The phase shifts of the two coils in the DCPD counteract each other, whereas, in the DCSFW, the phase shifts of the two coils are superimposed, thus increasing the phase shift of the output signal. The DCPD excitation method should be used for bi-directional displacement detection to achieve better signal modulation.

**Figure 16:**
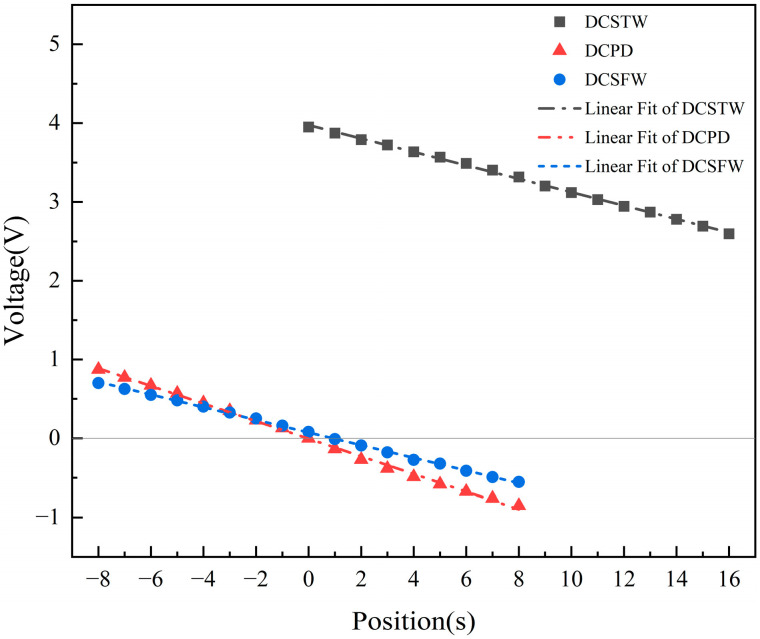
Comparison of the output signal with the three excitation methods (after filtering).

The output signal of the AD630 circuit is filtered by a low-pass filter, and the final DC voltage signal is output. The simulation results are shown in Figure 16.

As can be seen in Figure 16, the DCSTW has a linearity of 1.85% and a sensitivity of 84.75 mV/mm. The DCPD has a linearity of 2.80% and a sensitivity of 107.63 mV/mm. The DCSFW has a linearity of 2.02% and a sensitivity of 78.44 mV/mm. Therefore, DCSTW has better linearity, and DCPD has better sensitivity of the three excitation methods.

## 5. Excitation Method for Dual-Coil Inductive Displacement Transducers

### 5.1. Experimental Program Design

**Figure 17:**
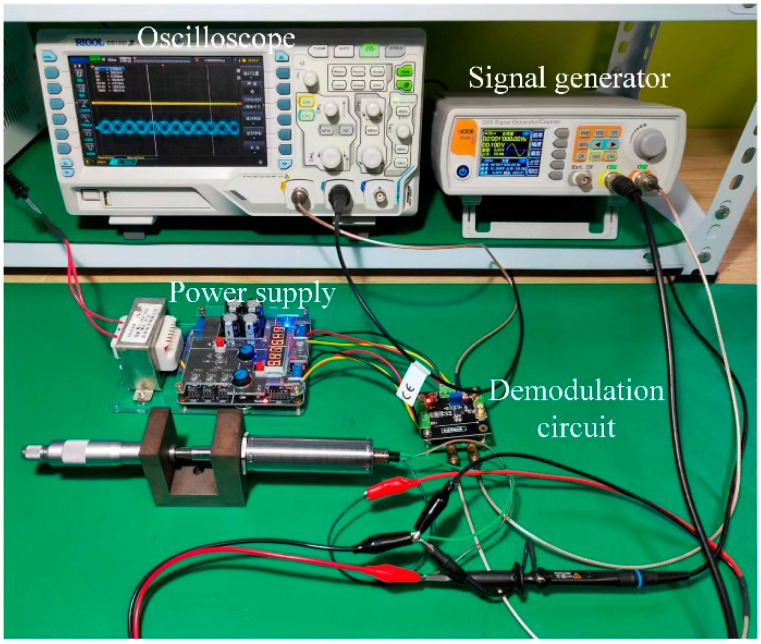
Experimental test.

Based on circuit simulations, the program’s effectiveness was verified by experiments. The generator provided an excitation signal to DIPT16 and a modulation signal to the conditioning circuit. The conditioning circuit was powered by an adjustable power supply, and the demodulation circuit output signal was tested using an oscilloscope. It should be noted that the conditioning circuit is a two-module structure, where module 1 consists of an operational amplifier, an AD630 circuit, and a low-pass filter, and module 2 is a differential circuit. This structure was adopted because the DCSTW does not require differential circuits, and the DCPD and DCSFW do. Therefore, the DCSTW was tested using Module 1, and the DCPD and DCSFW were tested using both Modules. There were some differences between the experimental tests and the simulation. To reduce external interference, the operational amplifier was tuned with adjustable resistors. The experimental tests are shown in Figure 17.

### 5.2. Results of Experimental Tests

**Figure 18:**
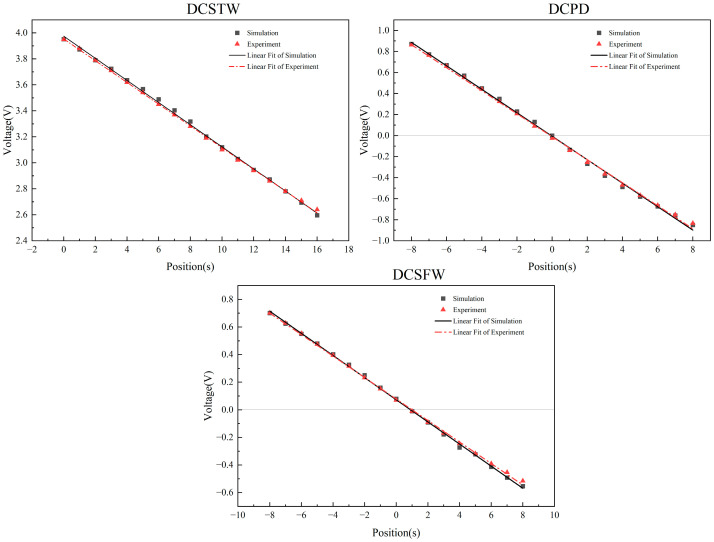
Simulation and experimental comparison of the three excitation methods.

In the three excitation methods, the DIPT16 design had the same conditioning circuit as the simulation. The inductive core displacement was adjusted to test the relationship between different displacements and the output signal and compared with the simulation results, as shown in Figure 18.

As seen in Figure 18, the DCSTW has a linearity of 1.93% and a sensitivity of 81.88 mV/mm. The experiments show a 0.07% reduction in linearity and a 2.87 mV/mm reduction in sensitivity compared to the simulation. The DCPD has a linearity of 3.0% and a sensitivity of 106.06 mV/mm. The experiments show a 0.2% reduction in linearity and a 1.57 mV/mm reduction in sensitivity compared to the simulation. The DCSFW has a linearity of 2.34% and a sensitivity of 76.06 mV/mm. The experiments show a 0.32% reduction in linearity and a 2.38 mV/mm reduction in sensitivity compared to the simulation. The ideal simulated circuit causes the difference between the experiment and the simulation. In practice, the components of the experimental circuit are inaccurate, and there are disturbances in the operation of the circuit.

From the above, it can be seen that the proposed excitation methods DCSTW and DCSFW have better linearity and sensitivity compared to commonly used excitation methods. The proposed coil excitation method is helpful to improve the performance of the sensor. The coil excitation method and demodulation circuit designed in this paper can be applied to industrial production and can improve measurement accuracy.

The experiments show that DCSTW has the best linearity, which is 35.67% higher than DCPD and 17.52% higher than DCSFW. The DCPD was highest in sensitivity, 29.53% higher than the DCSTW and 39.44% higher than the DCSFW. The simulation results show that the DCPD has the smallest phase shift, and the DCSFW has the largest phase shift. It is evident that the sensitivity is positively related to the phase shift and can be improved by reducing it.

There were certain errors in testing and simulation because the simulation was an ideal condition, while the components of the test were not ideal components, and there were experimental errors. There are several reasons for the errors. First, there was an error between the nominal value and the actual value of electronic components. Second, there was a measurement error made by the measurement personnel during the testing process. Third, there was external interference in the test environment. During testing, these errors are inevitable but can be minimized.

## 6. Conclusions

This paper analyzes the working principle and characteristics of a dual-coil inductive displacement transducer. It uses a conditioning circuit with the AD630 chip as the core to process the output signal of the displacement transducer. Based on the displacement transducer DIPT16, three coil excitation methods are proposed and investigated by simulation and experiment, leading to the following conclusions:

(1) A conditioning circuit with the AD630 chip as the core enables the demodulation of the output signal of a dual-coil inductive displacement transducer. Three excitation methods are proposed, namely DCSTW, DCSFW, and DCPD. The influence of three excitation methods on the performance of the transducer is analyzed.
(2) Experiments show that DCSTW can only perform single-direction displacement testing; DCPD and DCSFW can perform bi-directional displacement testing. DCSTW has maximum amplitude variation and optimal linearity. The linearity of DCSTW is the best, 35.67% higher than DCPD and 17.52% higher than DCSFW. DCPD has the highest sensitivity, 29.53% higher than DCSTW and 39.44% higher than DCSFW. DCPD has minimum phase shift and optimal sensitivity. The sensitivity is positively correlated with the phase shift, and can be improved by reducing the phase shift.
(3) In this paper, the working mechanism of the dual-coil inductive displacement transducer and the demodulation principle of the transducer are analyzed in depth. A suitable adjustment circuit is provided, which provides the basis for selecting an appropriate coil excitation method. The results of this study are beneficial to improving the performance of the dual coil displacement transducer.

## Figures and Tables

**Table 1:** The linear fit results of the coil inductance.

Equation	y = a + bx	
Plot	Coil 1	Coil 1
Weight	No Weighting	
Intercept	16.04207 ± 0.0781	29.88891 ± 0.10094
Slope	0.87012 ± 0.00839	−0.92165 ± 0.01052
Residual Sum of Squares	1.63189	2.89542
Pearson’s r	0.99856	−0.99792
R-Square (COD)	0.99713	0.99585
dj. R-Square	0.99703	0.99572

## Data Availability

The data presented in this study are available on request from the corresponding author.

## References

[1] Babu A., George B. (2016). Design and Development of a New Non-Contact Inductive Displacement Sensor. IEEE Sens. J..

[2] Ren Y.J., Zhao G.F., Qian M., Feng Z.H. (2019). A highly sensitive triple-coil inductive debris sensor based on an effective unbalance compensation circuit. Meas. Sci. Technol..

[3] Sandra K.R., Kumar A.S.A., George B., Kumar V.J. (2019). A Linear Differential Inductive Displacement Sensor With Dual Planar Coils. IEEE Sens. J..

[4] Grima A., Di Castro M., Masi A., Sammut N. (2020). Design Enhancements of an Ironless Inductive Position Sensor. IEEE Trans. Instrum. Meas..

[5] Grima A., Di Castro M., Masi A., Sammut N. Frequency response characterization of ironless inductive position sensors with long cables. Proceedings of the 3rd ICMIE.

[6] Jiao D., Ni L., Zhu X., Zhe J., Zhao Z., Lyu Y., Liu Z. (2019). Measuring gaps using planar inductive sensors based on calculating mutual inductance. Sens. Actuator APhys..

[7] Mirzaei M., Ripka P., Chirtsov A., Grim V. (2020). Temperature stability of the transformer position transducer for pneumatic cylinder. J. Magn. Magn. Mater..

[8] Liu R., Bu H. (2013). Design on LVDT displacement sensor based on AD598. Sens. Transducers.

[9] Reverter F., Gasulla M. (2017). Timer-Based Demodulator for AM Sensor Signals Applied to an Inductive Displacement Sensor. IEEE Trans. Instrum. Meas..

[10] Lu X., Tian G., Wang Z., Li W., Yang D., Li H., Wang Y., Ni J., Zhang Y. (2022). Research on the Time Drift Stability of Differential Inductive Displacement Sensors with Frequency Output. Sens..

[11] Li W., Hu J., Su Z., Hu J., Wang D. (2022). Analysis and design of axial inductive displacement sensor. Measurement.

[12] Yang Z., Song J., Cai W., Lu G., Zhang Z. (2021). Analysis of the influence of a solenoid magnetic field in the azimuth transmission system. Sci. Rep..

[13] Petrone C., Sorti S., Dalane E., Mehl B., Russenschuck S. (2022). Induction-coil measurement system for normal-and superconducting solenoids. IEEE Trans. Appl. Supercond..

[14] He Q., Fan S., Chen N., Tan R., Chen F., Fan D. (2021). Analysis of Inductive Displacement Sensors with Large Range and Nanoscale Resolution. Appl. Sci..

[15] Ozaki M., Yagitani S., Takahashi K., Imachi T., Koji H., Higashi R. (2015). Equivalent Circuit Model for the Electric Field Sensitivity of a Magnetic Search Coil of Space Plasma. IEEE Sens. J..

[16] Ren Z., Li H., Yu W. (2021). Research on Coil Impedance of Self-Inductive Displacement Sensor Considering Core Eddy Current. Sensors.

[17] Liu D., Zhang H., Xiao X., Ma Q., Li H., Tian G., Gao B., Wu J. (2022). RLC Parameters Measurement and Fusion for High-Sensitivity Inductive Sensors. IEEE Trans. Instrum. Meas..

[18] Braun T., Reuter J., Rudolph J. Position Observation for Proportional Solenoid Valves by Signal Injection. Proceedings of the 7th IFAC Symposium on Mechatronic Systems, Loughborough University.

[19] Kumar P., George B., Kumar V.J. A simple signal conditioning scheme for inductive sensors. Proceedings of the 2013 Seventh ICST.

[20] Djuric S.M., Djuric N.M., Damnjanovic M.S. (2015). The optimal useful measurement range of an inductive displacement sensor. Inf. Midem J. Microelectron. Electron. Compon. Mater..

[21] Xu X., Suganuma Y., Dhirani A.-A. (2020). Low-Cost, High-Performance Lock-in Amplifier for Pedagogical and Practical Applications. J. Chem. Educ..

[22] Chen D., Yang W., Pan M. (2011). Design of Impedance Measuring Circuits Based on Phase-Sensitive Demodulation Technique. IEEE Trans. Instrum. Meas..

[23] Nagarajan P.R., George B., Kumar V.J. (2017). A Linearizing Digitizer for Wheatstone Bridge Based Signal Conditioning of Resistive Sensors. IEEE Sens. J..

[24] Drumea A., Svasta P., Blejan M. Modelling and simulation of an inductive displacement sensor for mechatronic systems. Proceedings of the 33rd International Spring Seminar on Electronics Technology.

[25] Liu Y.-T., Zhang K., Xu Y. Inductive Displacement Sensors Based on the Integrated Demodulation Chip. Proceedings of the 4th International Symposium on Software Reliability, Industrial Safety, Cyber Security and Physical Protection for Nuclear Power Plant.

[26] Gal-Katziri M., Hajimiri A. (2019). Analysis and Design of Coupled Inductive Bridges for Magnetic Sensing Applications. IEEE J. Solid-State Circuit.

[27] Hu J., Li W., Su Z., Wang D. (2021). Analytical design and experiment of radial inductive displacement sensor with full-bridge structure for magnetic bearings. Rev. Sci. Instrum..

[28] Chattopadhyay S., Bera S.C. (2010). Modification of the Maxwell–Wien bridge for accurate measurement of a process variable by an inductive transducer. IEEE Trans. Instrum. Meas..

[29] Wang K., Zhang L., Le Y., Zheng S., Han B., Jiang Y. (2017). Optimized Differential Self-Inductance Displacement Sensor for Magnetic Bearings: Design, Analysis and Experiment. IEEE Sens. J..

[30] Gunasekaran V., George B., Aniruddhan S., Janardhanan D.D., Palur R.V. (2019). Performance Analysis of Oscillator-Based Read-Out Circuit for LVDT. IEEE Trans. Instrum. Meas..

[31] Reinholz D. (2022). A Novel Wide-Bandwidth Linear Inductive Differential Position Sensor. Master’s Thesis.

[32] Li H., Ren Z., Chen R., Yu W. (2022). Influence of structural parameters on the static performance of self-inductive radial displacement sensor. Meas. Sci. Technol..

[33] Guo Y.-X., Lai C., Shao Z.-B., Xu K.-L., Li T. (2019). Differential Structure of Inductive Proximity Sensor. Sensors.

